# Preliminary Phytochemical Composition and *In Vitro* Anthelmintic Activity of Aqueous and Ethanol Extracts of *Olea africana* against Mixed Gastrointestinal Worms in Dogs

**DOI:** 10.1155/2022/5224527

**Published:** 2022-08-16

**Authors:** Kenneth Otieno Orengo, James Mucunu Mbaria, Maingi Ndichu, Kitaa Jafred, Mitchel Otieno Okumu

**Affiliations:** ^1^Department of Public Health, Pharmacology and Toxicology, University of Nairobi, P. O. Box 29053-00200, Nairobi, Kenya; ^2^Department of Veterinary Pathology, Microbiology, and Parasitology, University of Nairobi, P. O. Box 29053-00200, Nairobi, Kenya; ^3^Department of Clinical Studies, University of Nairobi, P. O. Box 29053-00200, Nairobi, Kenya

## Abstract

*Olea africana* is used by some indigenous communities in Kenya to control gastrointestinal worms in animals. Plant-based anthelmintics are gaining popularity globally in the control of gastrointestinal worms in animals. The egg hatch inhibition assay was used to assess the *in vitro* anthelmintic efficacy of aqueous and ethanol leaf extracts of *O. africana* against the eggs of mixed gastrointestinal helminths in dogs. Probit regression was used to calculate the concentration of extracts that inhibited egg hatching by 50% (IC_50_). Albendazole was used as a control. Standard techniques were used to quantify the phytochemicals in the extracts. The aqueous extract had an IC_50_ of 1.85 mg/mL (1.64–2.10), and the ethanol extract had an IC_50_ of 0.25 mg/mL (0.23–0.26). Quantitative phytochemical analysis revealed that aqueous and ethanol extracts of *O. africana* contained alkaloids (19.40 and 61.60%), saponins (24.00 and 6.00%), phenols (0.95 and 1.28 mg/g gallic acid equivalents (GAE)), flavonoids (8.71 and 12.26 mg/g catechin equivalents (CE)), and tannins (67.30 and 76.30 mg/g of tannic acid equivalent (TAE)), respectively. *O. africana* has dose-dependent anthelmintic effects against mixed gastrointestinal worms in dogs. These findings support the traditional use of *Olea africana* as a treatment option for gastrointestinal worms in dogs.

## 1. Introduction

Helminths are parasitic worms that include nematodes, cestodes, and trematodes [1]. They are a major public health concern in many parts of the world [1]. Anthelmintics are used to control these worms, but poor sanitation and a lack of effective vaccines in helminth-endemic areas around the world have limited the efforts to disrupt their transmission [2–4]. There are only a few anthelmintics on the market to combat parasitic worms [5, 6]. Resistance to available anthelmintics is also a persistent issue [5, 7–11]. These factors highlight the importance of developing alternative helminth treatment options.

Plant-based anthelmintics are gaining popularity globally in controlling gastrointestinal worms in animals [12–15]. The aqueous leaf extract of *Annona muricata* showed 84.91% and 89.08% of efficacy in the egg hatch inhibition and larval motility tests, respectively, and totally immobilized worms in the first 8 hours of nematode exposure [12]. The aqueous and ethanol extracts of *Thymus capitatus* completely inhibited *Haemonchus contortus* egg hatching at low concentrations, with the ethanol extract showing higher *in vitro* activity (paralysis/death) against adult parasites than the aqueous extract at different time intervals post-treatment [14]. Mazhangara and colleagues reported dose- and time-dependent inhibitory effects of some extracts of *Elephantorrhiza elephantina* against adult *Paramphistomum cervi* worms in goats [15].

*Olea africana* is a shrub or a small to medium-sized tree that grows to a height of between 5 and 10 meters [16]. It has a grey bark, narrow elliptical leaves, and green, grey, or yellow flowers. It is distributed in India, Kenya, Tanzania, Uganda, Eritrea, Ethiopia, Zimbabwe, Swaziland, Somalia, and South Africa [16]. Many names have been given to the plant, including kau, kao, brown olive, weira (Amharic), Olbaum (German), wera (Somali), bair banj (Hindi), and zeitun bari (Arabic) [16].

*Olea africana* has been reported to have antimalarial, antioxidant, antibacterial, and cardioprotective effects against doxorubicin toxicity [17–20]. Additionally, ethnopharmacological reports from Kenya show that pastoralist communities use *Olea africana* to treat gastrointestinal conditions [21, 22]. However, these claims have yet to be scientifically validated. As a result, the present study has two objectives: (1) to determine the *in vitro* anthelmintic activity of aqueous and ethanol leaf extracts of *Olea africana* against mixed gastrointestinal helminths in dogs and (2) to determine the preliminary phytochemical composition of aqueous and ethanol leaf extracts of *Olea africana*.

## 2. Methods

### 2.1. Ethical Considerations

The institutional ethics committee was consulted before the initiation of this study. **REF: FVM BAUEC/2019/199**. See Supplementary Materials, where the name of the ethics committee, date of ethical approval, and other relevant details on the approval are highlighted.

### 2.2. Collection of Plant Materials

Fresh leaves of *Olea africana* were collected in their natural habitat near Loosuk Village in Loosuk Ward, Samburu West Sub-County of Samburu County. Other plant parts were collected and submitted to the Kenya National Museums Herbarium for botanical identification. The leaves were air dried and ground to a fine powder, which was then kept in a cool place devoid of moisture.

### 2.3. Preparation of the Aqueous Extract of *O. africana*

The method described in a previous publication was employed [23]. Dry powder of *O. africana* was mixed with 900 mL of distilled water in a round-bottomed flask and heated at 100°C for 15 minutes. The mixture was cooled, centrifuged, filtered, and freeze-dried before the percentage yield was calculated [23].

### 2.4. Preparation of the Ethanol Extract of *O. africana*

The method of Were et al. was used [24]. To enhance extraction, two hundred grams of *O. africana* dry powder was mixed with 1000 mL of ethanol (Fisher Scientific, UK) in a round-bottomed flask for 72 hours with regular shaking before filtration and rotary evaporation [24]. The extract's percentage yield was then calculated [24].

### 2.5. Preparation of Extract Stock Solutions and Serial Dilutions

Stock solutions of the extracts of *O. africana* were prepared by vortexing (Digisystem, Taiwan) half a gram of the extracts in 5 mL of distilled water. In microtiter plates, each extract was subjected to a 16-point serial dilution. One hundred microliters (100 *µ*L) of distilled water was pipetted into the 16-well microtiter plate (A-H for two columns of a microtiter plate). 180 *µ*L of the stock solution was pipetted and mixed into the first well (A of 1^st^ column). An equal volume (180 *µ*L) was pipetted from the first well to the second well. This was repeated until the 16^th^ well, with each dilution lowering the concentration by 0.9.

### 2.6. Experimental Animals

For this study, disease-free dogs (pups) (*n* = 3) aged 8–10 weeks with no history of deworming were used. The animals were housed in clean and disinfected kennels and immunized against canine parvovirus, canine distemper, canine hepatitis, and leptospirosis and fed for one week before fecal samples were collected (acclimatization). The natural infestation was detected using fecal smears. After that, pooled fecal samples were collected.

### 2.7. Preparation of Egg Solution

The fecal flotation technique described by Blagburn and Butler was used to collect fecal samples [25]. Fresh pooled fecal samples weighing 100 to 200 grams were collected from the animals in the morning before the *in vitro* experiments were set up. The fecal material was homogenized with 200 mL of saturated salt solution [25]. Using a plastic sieve, the mixture was filtered into 100 mL measuring cylinders, and the filtrate was used to fill the cylinders to the 100 mL mark. To make contact with the fluid meniscus at the top of the measuring cylinder, a clean glass slide was placed on the glass slide. This setup was allowed to stand for ten minutes [25]. The glass slides were removed, and the surfaces in contact with the fluid meniscus were washed in glass Petri dishes with distilled water. This was repeated to recover as many eggs as possible. The egg solution from the Petri dishes was collected and transferred to 50 mL peak-bottomed plastic centrifuge tubes. The solution was centrifuged, and the supernatant discarded before distilled water was added and vortexed. The resulting mixture was centrifuged to get rid of as much salt as possible [25]. The egg solution was obtained by adding 10 mL of distilled water to the final sediment and shaking it with a vortex mixer. Using the McMaster technique, the number of eggs in 50 *µ*L of the egg solution was counted. The counting was repeated three times using three different slides to determine the average number of eggs in the egg solution, which varied depending on the sampling day [25].

### 2.8. The Egg Hatch Inhibition Assay (EHIA)

The method described by Coles et al. was used [26]. In brief, 100 *µ*L of serial dilutions of the extracts (0.7–6 mg/mL) were pipetted into 96-well microtiter plates. Egg solution (100 *µ*L) was pipetted into extract-containing wells. Distilled water (100 *µ*L) was the negative control, while 0.25 mg/mL of albendazole solution in distilled water was the positive control. The plate was labelled, covered, and wrapped in foil before being incubated in a humidified incubator at 37°C for 72 hours [26]. Each well was examined for free larvae under an inverted microscope at 10× magnification. Hatched and unhatched eggs from each well were counted and recorded. Triplicate determinations were made.

### 2.9. Qualitative Phytochemical Screening

The secondary metabolites present in the extracts of *O. africana* were evaluated using standard techniques [27, 28].

#### 2.9.1. Evaluation of Alkaloids

0.05 g of each extract was mixed with distilled water and hydrochloric acid (Rankem, India). The resulting mixture was filtered, and the filtrate was transferred to a test tube. Dragendorff's reagent was added, and the colour changes were observed and reported.

#### 2.9.2. Evaluation of Anthraquinones

0.005 g of each extract was mixed with 10 mL of benzene (Rankem, India) and filtered into a test tube, and the filtrates were mixed with 10% ammonia solution (Rankem, India). The colour changes were observed and reported.

#### 2.9.3. Evaluation of Cardiac Glycosides

0.5 g of each extract was mixed with water, glacial acetic acid (Rankem, India), ferric chloride solution (FeCl_3_) (FINAR, India), and concentrated sulphuric acid (H_2_SO_4_) (Rankem, India). The colour changes were observed and reported.

#### 2.9.4. Evaluation of Flavonoids

Five drops of 5% sodium hydroxide (NaOH) (Rankem, India) and 2 M hydrochloric acid (HCl) (Rankem, India) were mixed with each extract, and the colour change was observed and reported.

#### 2.9.5. Evaluation of Phenolics (Ferric Chloride Test)

Distilled water and 10% aqueous ferric chloride solution (FeCl_3_) (FINAR, India) were added to 0.0001 g of the extracts. The colour changes were observed and reported.

#### 2.9.6. Evaluation of Saponins

Distilled water was mixed with each of the extracts in a test tube and shaken well for a period of 5 minutes. Foam formation was observed and reported.

#### 2.9.7. Evaluation of Tannins

Distilled water and 5% ferric chloride solution (FeCl_3_) (FINAR, India) were mixed with each of the extracts. The colour changes were observed and reported.

#### 2.9.8. Test for Triterpenes (Salkowski's Test)

0.0002 g of the extracts was shaken with 1 mL of chloroform (CHCl_3_) (LOBA Chemie, India). A few drops of concentrated sulphuric acid (H_2_SO_4_) (Rankem, India) was added, and the colour changes were observed and reported.

### 2.10. Quantitative Phytochemical Screening

Standard methods for quantifying phytochemicals such as phenolics [29, 30], flavonoids [31], tannins [32], saponins [33, 34], and alkaloids [27] were employed.

#### 2.10.1. Total Phenolic Content

Standard methods [29, 30] were used with modifications [35]. Gallic acid (LOBA Chemie, India) was used as the standard. A stock solution was prepared by dissolving 0.01 g of gallic acid in 100 mL of methanol. Different volumes of this solution ranging from 0.25 to 2 mL were pipetted into 5 different 10 mL volumetric flasks and 2.5 mL of a 1 : 10 v/v Folin phenol reagent (LOBA Chemie), and distilled water was added followed by 2.0 mL of a 7.5% w/v sodium carbonate (Na_2_CO_3_) (LOBA Chemie) solution. Distilled water was added to the 10 mL mark. The blank was prepared by mixing the aforementioned reagents and water. Test samples and blank were kept in a water bath at 45°C for 15 minutes for colour development. The resulting mixtures were transferred to suitable cuvettes, and the absorbance was measured at 765 nm using a spectrometer (Milton Roy, USA). A standard calibration curve was prepared. Each extract was prepared by dissolving 0.01 g of the extracts in 10 mL methanol (Fisher Scientific, UK). Some of this solution (1 mL) was pipetted to a 10 mL volumetric flask, and the procedure followed in preparing the standard curve was followed. Triplicate determinations were made, and the final results were summarized using averages that were used to infer the concentrations from the standard curve. The formula described by Gouveia and Castilho [36] was used to calculate the phenolic content:

total phenolic content = concentration of gallic acid established from the calibration curve × volume of the extract/weight of the extract.

#### 2.10.2. Total Flavonoid Content

The method of Atanassova et al. [31] was used with modifications [35]. Catechin (FINAR, India) was used as the standard. A stock solution was prepared by dissolving 0.01 g of catechin in 100 mL of methanol (Fisher Scientific, UK). 4 mL of distilled water, 0.3 mL of sodium nitrite (LOBA Chemie), 0.3 mL of aluminium chloride (FINAR, India), and 2 mL of 1 M sodium hydroxide (NaOH) (Rankem, India) were mixed with different volumes of the stock solution ranging from 0.1 to 1.0 mL. The volumetric flasks were made up to 10 mL with distilled water and transferred into cuvettes, and the absorbance was measured at 510 nm using a spectrometer (Milton Roy, USA). Distilled water was used as the reagent blank, and a calibration curve was prepared. 0.01 g of each extract was dissolved in 10 mL methanol (Fisher Scientific, UK), and 1 mL of this was pipetted into a 10 mL volumetric flasks containing 4 mL of distilled water, 0.3 mL of 5% w/v sodium nitrite (NaNO_2_) (LOBA-Chemie), 0.3 mL of 10% w/v aluminium chloride (AlCl_3_) (FINAR, India), and 2 mL of 1 M sodium hydroxide (NaOH) (Rankem, India). The mixture was then made up to the mark with distilled water. The resulting mixture was transferred into cuvettes, and the absorbance was taken at 510 nm using a spectrometer (Milton Roy, USA). Distilled water was the reagent blank. Triplicate determinations were made, and the results were averaged and used to evaluate the concentration of flavonoids in the extracts using the formula described by Gouveia and Castilho [36]:

total flavonoid content = concentration of catechin based on the calibration curve × volume of the extract/weight of the extract.

#### 2.10.3. Saponin Content

Saponin content was determined using the methods of Ejikeme et al. [33] and Obadoni and Ochuko [34]. The process involved mixing 100 mL of 20% aqueous ethanol (Fisher Scientific, UK) and the extracts in a 250 mL conical flask. A water bath (Memmert, Germany) was used to heat the mixture at 55°C for 4 hours. The mixture was filtered and the residue was collected, extracted with ethanol, and rotary evaporated. 20 mL of diethyl ether (LOBA Chemie, India) was mixed with the concentrate in a 250 mL separating funnel. The aqueous layer was collected, and the process was repeated before 60 mL of n-butanol (LOBA Chemie, India) and 10 mL of 5% w/v sodium chloride (Rankem, India) were added. The sodium chloride layer was discarded, and the residual solution was heated in a water bath for half an hour before being transferred into a crucible and dried in an oven. The saponin content was calculated as a percentage using the formula below [37]:

(1) $$\begin{array}{c} \%\text{ Saponin}=\frac{\text{Weight of Saponin}}{\text{Weight of sample}}\times 100. \end{array}$$

#### 2.10.4. Alkaloid Content

The method of Harborne was used [27]. 200 mL of 10% acetic acid in ethanol (LOBA Chemie) was mixed with 2.5 grams of each extract in a 250 mL beaker. The mixture stood for 240 minutes before being concentrated in a water bath. 15 drops of concentrated ammonium hydroxide (NH_4_OH) (Sigma Aldrich, USA) was added dropwise to the concentrate and stood for 3 hours, then the supernatant was discarded, and the precipitates were washed with 20 mL of 0.1 M of ammonium hydroxide (NH_4_OH) (Sigma Aldrich, USA) and filtered. The residue was transferred to a crucible and dried in an oven [27].

(2) $$\begin{array}{c} \text{The percentage of alkaloid in the sample was expressed as }\%\text{ alkaloid}=\frac{\text{Weight of alkaloid}}{\text{Weight of sample}}\times 100. \end{array}$$

#### 2.10.5. Tannin Content

The methods of Amadi and others [32] and Ejikeme and colleagues [33] were used. The Folin-Denis reagent was mixed with phosphomolybdic acid (H_3_PMo1_2_O_4_0) (LOBA Chemie, India) and orthophosphoric acid (H_3_PO_4_) (BDH-Prolabo, Dubai) and refluxed for two hours. Distilled water was added followed by tannic acid (LOBA Chemie, India), and the mixture was pipetted into a 200 mL volumetric flask. Distilled water was used to make up the resulting mixture to 250 mL. Concentrations of the standard solution ranging from 0.2 to 1.0 mg/mL (LOBA Chemie, India) were pipetted into 25 mL volumetric flasks, and 1.25 mL of Folin-Denis reagent (FINAR, India) was added followed by 2.5 mL of Na_2_CO_3_ (FINAR, India) solution. The mixture was made up to 100 mL with distilled water and kept in a water bath maintained at 25°C for half an hour. The optical density was measured using a spectrometer (Milton Roy, USA) at 700 nm, and a standard curve was plotted. 0.5 grams of each extract was transferred into a conical flask and dissolved in 50 mL of distilled water. This mixture was boiled for 1 hour on an electric hot plate and filtered. 2.5 mL of Folin-Denis reagent (FINAR, India), 5 mL of saturated Na_2_CO_3_ solution (FINAR, India), and 25 mL of distilled water were mixed with 5 mL of the diluted extract. The solution was kept stand in a water bath at 25°C for half an hour. Absorbance was measured at 700 nm using a spectrometer (Milton Roy, USA). The formula described by Sheikh et al. was used to calculate the tannic acid content in the extracts [38]:

(3) $$\begin{array}{c} \text{Tannic acid}\left(\frac{\text{mg}}{100g}\right)=\frac{\text{Concentration of tannic acid from the graph}\times \text{extract volume}\times 100}{\text{Aliquot volume}\times \text{weight of sample}}. \end{array}$$

### 2.11. Data Analysis

The percentage inhibition of eggs was calculated using equation (4) as described by Coles and colleagues [26]:

(4) $$\begin{array}{c} \text{Percentage Inhibition}\left(\%\right)=1-\frac{P\text{ Test}}{P\text{ Untreated }}x100, \end{array}$$

where *P* Test = percentage inhibition of eggs in extract-treated wells/positive control and *P* Untreated = percentage of eggs inhibited wells where no treatment was applied.

The concentration of extracts responsible for 50% inhibition of egg hatching (EC_50_) was calculated using probit regression analysis on the IBM Statistical Package for the Social Sciences (IBM) [39]. The differences in the dose-response effects of the extracts on egg hatching were assessed on GraphPad Prism (9.0.0) using one-way ANOVA. Multiple comparisons were made using Tukey's post hoc tests. *P* < 0.05.

## 3. Results

The percentage yield of *O. africana* aqueous and ethanol extracts was 2.50% w/w and 12.00% w/w, respectively (Table 1). The aqueous extract was dark brown, whereas the ethanol extract was dark green and mostly made of sticky granules (Table 1).

**Figure 1:**
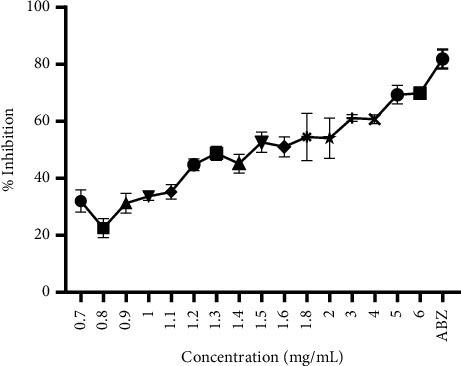
Plot of the effect of the aqueous extract of *Olea africana* and albendazole on egg hatching. The error bars represent the standard deviation of triplicate measurements of the % inhibition at each concentration.

Figure 1 depicts how different doses of the aqueous extract of *Olea africana* and albendazole affect egg hatching.

In general, the higher the dose of the aqueous extract, the more pronounced the inhibition of egg hatching (Figure 1). The inhibitory capacity of albendazole against egg hatching was greater than that of graded doses (0.7 mg/mL to 6 mg/mL) of the aqueous extract of *Olea africana* (Figure 1).

**Figure 2:**
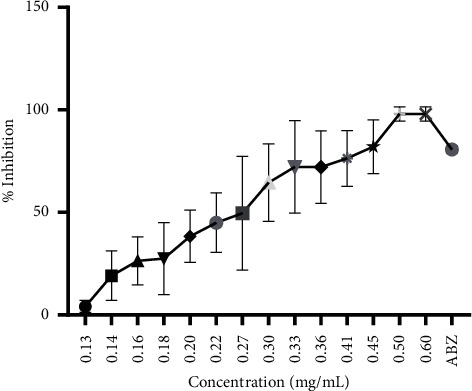
Plot of the effect of the ethanol extract of *Olea africana* and albendazole on egg hatching. The error bars represent the standard deviation of triplicate measurements of the % inhibition at each concentration.

Figure 2 depicts how different doses of the ethanol extract of *Olea africana* and albendazole affect egg hatching.

The EC_50_ values of aqueous and ethanol extracts of *Olea africana* against mixed gastrointestinal worms of dogs are shown in Table 2. The EC_50_ of the aqueous extract was 1.85 mg/mL (1.64–2.10), while the EC_50_ of the ethanol extract of *Olea africana* was 0.25 mg/mL (0.23–0.26) (Table 2).

The preliminary phytochemical screening of aqueous and ethanol extracts of *O. africana* is shown in Table 3. Anthraquinones, terpenoids, saponins, glycosides, flavonoids, phenolics, and tannins were identified in the aqueous extract, while alkaloids, terpenoids, flavonoids, glycosides, phenolics, saponins, and anthraquinones were discovered in the ethanol extract.

The quantitative phytochemical composition of the aqueous and ethanol extracts of *O. africana* is shown in Table 4. The flavonoid, phenolic, and saponin contents of the ethanol extract of *O. africana* were higher than those of the aqueous extract of *O. africana* (Table 4).

## 4. Discussion

Different aspects of the helminth's life cycle, such as egg hatching, larval motility, and adult parasites, are targeted in evaluating the *in vitro* anthelminthic efficacy of various compounds [40]. The current study used *Olea africana* extracts to target egg hatching of mixed gastrointestinal worms from dogs. In the present study, the ethanol extract of *Olea africana* was more effective than the aqueous extract. An investigation into the effect of different solvents (ethanol, dichloromethane, water) on the anthelmintic activity of *Warburgia salutaris*, *Allium sativum, Ananas comosus, Aloe ferox,* and *Lespedeza cuneata* revealed that the ethanol extract was the most effective solvent with larval counts decreasing with increasing extract concentrations of which 10% and 20% had similar effects [41]. Furthermore, the percentage of egg hatch inhibition by the aqueous extract ranged from 22.54% to 69.87%. In comparison, the percentage of egg hatch inhibition by the ethanol extract ranged from 4.10% to 98.02%, indicating a dose-response relationship. A dose-response relationship ranging from 18% to 42% was also observed when *Haemonchus contortus* from sheep was exposed to the aqueous and methanol extracts of *Euphorbia helioscopia* [13]. A drug/agent is considered an effective anthelmintic if it inhibits egg hatching by more than 90% [42]. When this is taken into account, it is possible to argue that the ethanol extract of *O. africana* is effective at doses greater than 0.5 mg/mL. Indeed, when the *in vitro* results of both extracts were compared side by side using the dose range of 0.7–6 mg/mL, it was observed that the ethanol extract inhibited egg hatching completely (100%). To calculate the EC_50_ value of this extract, the doses of the ethanol extract of *O. africana* had to be tapered down from 0.13 mg/mL to 0.6 mg/mL.

*Olea africana* aqueous and ethanol extracts contain anthraquinones, flavonoids, glycosides, phenolics, saponins, and tannins. Grunberg and Cleeland found that 1,4-bis(2-diethylamino ethoxy) anthraquinone dihydrochloride was more effective than quinacrine in clearing *Hymenolepis nana* from mice and piperazine in clearing *Syphacia obvelata* from naturally infected mice [43]. *Haemonchus contortus* eggs and infective larvae were reported to be sensitive to galloyl flavonoids derived from the pods of *Acacia farnesiana* [44]. *Achyranthes aspera* was reported to contain rutin, chlorogenic acid, and genistein (phenolic compounds) [45]. Dioscin and polyphyllin D (steroidal saponins) from the methanol extract of *Paris polyphylla* were more effective than mebendazole against *Dactylogyrus intermedius* [46]. Furthermore, purified condensed tannins were effective against free-living and parasitic stages of *Oesophagostomum dentatum* [47]. Based on our findings, it is unclear which of the phytoconstituents we identified was responsible for the observed anthelmintic activity. Further work may be needed to isolate and test molecules from the ethanol extract of *Olea africana* in order to identify the most pharmacologically active molecules.

## 5. Conclusions

*Olea africana* has dose-dependent anthelmintic effects against mixed gastrointestinal worms in dogs. These findings validate the traditional use of *Olea africana* as a treatment option for gastrointestinal worms in dogs.

## Figures and Tables

**Table 1:** Summary of the percentage yield and properties of *Olea africana* extracts.

Serial number	Sample extract	Percentage yield (%w/w)	Characteristics of the samples
1	Aqueous extract of *O. africana*	2.50	Dark brown crystals and hygroscopic powder
2	Ethanol extract of *O. africana*	12.00	Dark green sticky granules

**Table 2:** Summary of the EC_50_ values of aqueous and ethanol extracts of *Olea africana*.

Serial number	Sample extract	EC_50_ (*µ*g/mL)	95% CI
1	Aqueous extract of *O. africana*	1.85	1.64–2.10

2	Ethanol extract of *O. africana*	0.25	0.23–0.26

EC_50_: extract concentration responsible for 50% inhibition of egg hatching; CI: confidence interval.

**Table 3:** Qualitative phytochemical screening of aqueous and ethanol extracts of *Olea africana*.

Plant metabolite	Aqueous extract of *O. africana*	Ethanol extract of *O. africana*
Alkaloids	—	+
Anthraquinones	+	+
Flavonoids	+	+
Glycosides	+	+
Phenolics	+	+
Saponins	+	+
Tannins	+	+
Terpenoids	+	*P* < 0.05

**Table 4:** Quantitative phytochemical composition of the aqueous and ethanol extracts of *Olea africana*.

Sample	Total alkaloids (%)	TFC (mg/g GAE)	TPC (mg/g CE)	TAC (mg/g TAE)	Total saponin (%)
Aqueous	19.40	8.71	0.95	67.30	24.00
Ethanol	61.60	12.26	1.28	76.30	6.00

TFC: total flavonoid content; TAC: tannic acid content; TPC: total phenolic content; GAE: gallic acid equivalents; CE: catechin equivalents.

## Data Availability

The data used to support the findings of this study are available from the corresponding author upon request

## Supplementary Materials

Supplementary Materials The name of the ethics committee, date of ethical approval, and other relevant details on the approval are highlighted.
