# Supplementary material for: Preliminary Phytochemical Composition and In Vitro Anthelmintic Activity of Aqueous and Ethanol Extracts of Olea africana against Mixed Gastrointestinal Worms in Dogs
Source: Evid Based Complement Alternat Med. 2022 Aug 16;2022:5224527. doi: 10.1155/2022/5224527 (PMC9398766; doi:10.1155/2022/5224527)
Supplement: Supplementary Materials — The name of the ethics committee, date of ethical approval, and other relevant details on the approval are highlighted. [file 5224527.f1.pdf]

UNIVERSITY OF NAIROBI
FACULTY OF VETERINARY MEDICINE
DEPARTMENT OF VETERINARY ANATOMY AND PHYSIOLOGY

P.O. Box 30197,
00100 Nairobi,
Kenya.

Tel: 4449004/4442014/ 6
Ext. 2300
Direct Line. 4448648

Dr. Kenneth Orengo
University of Nairobi
Dept of PHP&T

**REF: FVM BAUEC/2019/199**

07/03/2019

Dear Dr. Orengo,

**RE: Approval of Proposal by Biosafety, Animal use and Ethics committee**

**Antihelmintic activity, phytochemistry and toxicity of selected medicinal plants on mixed gastrointestinal worms of dogs.**

**By Dr. Orengo J87/52287/2017.**

We refer to your PhD. proposal submitted to our committee for review and your application letter dated 28/02/2019.

We have reviewed your proposal, particularly section 3.5 that involves use of dogs and rats

We are satisfied that the proposed treatment and care of the animals meets acceptable standards for animal welfare. Furthermore, the numbers proposed are reasonable.

We have also noted that registered veterinary surgeons will supervise the work and humane end points.

We hereby give approval for you to proceed with the experiments as outlined in the submitted proposal.

Yours sincerely

Kaluwa

Dr. Catherine Kaluwa, BVM, MSc, Ph.D
Chairperson,
Biosafety, Animal Use and Ethics Committee
Faculty of Veterinary Medicine.
